# Supplementary material for: Observation of space-time surface plasmon polaritons
Source: Nat Commun. 2025 Dec 1;16:10697. doi: 10.1038/s41467-025-65289-8 (PMC12669788; doi:10.1038/s41467-025-65289-8)
Supplement: Supplementary file 1 — Description of Additional Supplementary Files [file 41467_2025_65289_MOESM1_ESM.pdf]

Description of Additional Supplementary Files

File Name: Supplementary Movie 1

Description: Time-resolved movie of the conventional SPP wave packet excited from a 5-µm-long nanoslit

Time-resolved movie of the conventional SPP wave packet excited from a 5-μm-long nano-slit at $z = 0$. The SPP wave packet propagates to the right (in the positive z -direction) while exhibiting intensity drop and diffractive spreading, with a faintly curved beat pattern. The focused spot size of the reference pulse is $\approx 60 \times 15\ \mu\mathrm{m}^2$, irradiating the right side near the nano-slit. The vertically elongated elliptical region observed at the nano-slit, which appears to shift in the negative z-direction as the delay progresses, arises due to the delay-dependent overlap position of the pump and reference pulses on the Ag surface.

File Name: Supplementary Movie 2

Description: Time-resolved movie of the conventional SPP wave packet with the extracted beat pattern

Movie of the field beat patterns extracted from the time-resolved movie of the conventional SPP wave packet (Supplementary Movie 1).

File Name: Supplementary Movie 3

Description: Time-resolved movie of the striped ST-SPP

Time-resolved movie of the striped ST-SPP excited from a nano-slit at $z = 0$ exhibits diffraction-free propagation over a distance of $\sim 100\ \mu\mathrm{m}$ ($> 4 \times z_\mathrm{R}$) while maintaining its checkered, lattice-like structure. The focused spot size of the reference pulse is $\approx 60 \times 15\ \mu\mathrm{m}^2$; the irradiation is horizontally elongated to enable long-range propagation monitoring under a constant reference condition. The acquisition time per frame is increased in the later part of the delay region to enhance the visibility of the SPP field.

File Name: Supplementary Movie 4

Description: Time-resolved movie of the striped ST-SPP with the extracted beat pattern

Movie of the field beat patterns extracted from time-resolved movie of the striped ST-SPP (Supplementary Movie 3).

File Name: Supplementary Movie 5

Description: Time-resolved movie of the subluminal ST-SPP near the nanoslit

Time-resolved movie of the subluminal ST-SPP observed near the nano-slit. The wave packet center, which has a characteristic lattice-like structure surrounded by tilted phase fronts, is visualized as it passes through the reference pulse irradiation, with a focused spot size of $\approx 36 \times 33\ \mu\mathrm{m}^2$ centered at $\sim 30\ \mu\mathrm{m}$ away from the nano-slit.

File Name: Supplementary Movie 6

Description: Time-resolved movie of the subluminal ST-SPP 80 µm away from the nanoslit

Time-resolved movie of the subluminal ST-SPP observed $\sim 80\ \mu\mathrm{m}$ away from the nano-slit. The reference pulse irradiated a circular area (focused spot size: $\approx 36 \times 33\ \mu\mathrm{m}^2$), centered at a distance of $\sim 80\ \mu\mathrm{m}$ from the nano-slit.
